# Effect of personalized dietary advice to increase protein intake on food consumption and the environmental impact of the diet in community-dwelling older adults: results from the PROMISS trial

**DOI:** 10.1007/s00394-022-02896-x

**Published:** 2022-07-05

**Authors:** Alessandra C. Grasso, Margreet R. Olthof, Ilse Reinders, Hanneke A. H. Wijnhoven, Marjolein Visser, Ingeborg A. Brouwer

**Affiliations:** Department of Health Sciences, Faculty of Science, and the Amsterdam Public Health Research Institute, Vrije Universiteit Amsterdam, Amsterdam, The Netherlands

**Keywords:** Older adults, Protein, Sustainability, Diet, RCT

## Abstract

**Purpose:**

Diet modelling studies suggest that increasing protein intake with no consideration for sustainability results in a higher environmental impact on the diet. To better understand the impact in real life, the aim of this study was to assess the effect of dietary advice to increase protein intake on food consumption and the environmental impact of the diet in community-dwelling older adults.

**Methods:**

Food consumption and environmental impact were analyzed among 124 Dutch older adults with lower habitual protein intake (< 1.0 g/kg adjusted body weight/day) participating in the six-month PROMISS trial. Dietary intake data from three 24-h dietary recalls, aided by food diaries, and results from life cycle assessments were used to examine the differences in changes in food consumption and environmental impact between those who received dietary advice to isocalorically increase protein intake to ≥ 1.2 g/kg aBW/d (Protein + ; *n* = 84) and those who did not receive dietary advice (Control; *n* = 40).

**Results:**

Compared to the Control, Protein + increased protein intake from animal-based food products (11.0 g protein/d, 95% CI 6.6–15.4, *p* < 0.001), plant-based food products (2.1 g protein/d, 95% CI 0.2–4.0, *p* = 0.031) and protein-enriched food products provided during the trial (18 g protein/d, 95% CI 14.5–21.6, *p* < 0.001) at the 6-month follow-up. Diet-associated greenhouse gas emissions increased by 16% (*p* < 0.001), land use by 19% (*p* < 0.001), terrestrial acidification by 20% (*p* = 0.01), and marine eutrophication by 16% (*p* = 0.035) in Protein + compared to the Control.

**Conclusion:**

This study found that dietary advice increased protein intake, favoring animal-based protein, and increased the environmental impact of the diet in older adults.

**Trial registration:**

ClinicalTrials.gov. NCT03712306. October 2018.

**Supplementary Information:**

The online version contains supplementary material available at 10.1007/s00394-022-02896-x.

## Introduction

While protein is a key component of the diet for optimal physical function throughout all life stages, it is of particular importance among older adults as they frequently experience a reduction in muscle mass and function [1]. As people age, physiological, psychological and environmental changes associated with reduced food consumption increase the risk of suboptimal protein intake [1–3]. Lower protein intake has been shown to be associated with sarcopenia [4, 5], which is characterized by a decline in muscle mass, strength, and physical function [6], increased risk of mortality and comorbidities [7, 8]. While several short-term metabolic and observational studies suggest that older adults aged 65 years and older need to consume more protein compared to younger adults to maintain adequate muscle mass and strength [9–13], evidence from randomized controlled trials is not conclusive [14]. Nevertheless, several expert groups propose an increase of the recommended daily allowance (RDA) of protein from 0.8 g/kg body weight (BW)/d to 1.0–1.2 g/kg BW/d for older adults [15–17].

It has been previously argued that increasing protein intake based on current food consumption patterns is likely to have unfavorable consequences for the natural environment [18–20]. Globally, current food production and consumption are dominant drivers of climate change, eutrophication (excessive richness of nutrients in water), acidification (water or soil becomes too acid), and biodiversity loss and are a considerable drain on resources such as land, water, energy, and nutrients [21, 22]. Notwithstanding the various components of the human diet, all of which have some impact on the environment, animal-based protein sources have been identified as having the largest impact on the environment than other dietary components [23, 24]. In the Netherlands, animal-based protein constitutes 60% of total protein consumed by young and older adults alike [25, 26]. A theoretical high-protein diet, modelled based on actual food intake data of Dutch community-dwelling older adults, showed that increasing protein intake from 1.0 to 1.2 g/kg BW/d, with isocaloric replacement and no consideration of environmental sustainability, increased the contribution of animal-based protein to 65% of total protein and increased diet-associated greenhouse gas emissions (GHGE) by 12–14%, land use (LU) by 10–12%, and fossil energy use by 9–10% [20]. This study used diet optimization, which is a powerful tool to model realistic diets as it simultaneously combines a given set of nutritional and environmental constraints while staying as close to the habitual diet as possible. However, given the diversity and complexity of food consumption behavior, understanding the effect of increasing protein intake on diet composition and diet sustainability in real life remains warranted.

Therefore, this study aimed to assess the change in consumption of protein-rich foods achieved by the PRevention Of Malnutrition In Senior Subjects in the EU (PROMISS) trial and to examine the effect of these changes over six months on the environmental impact of the diet. The PROMISS trial provides a unique opportunity to investigate the effect of the personalized dietary advice aiming at isocalorically increasing protein intake on food consumption and diet sustainability in community-dwelling older adults with low protein intake under real-life circumstances [27].

## Methods

### Study design and subjects

The PROMISS trial was a 6-month randomized controlled trial that investigated the effect of personalized dietary advice aiming at increasing protein intake with or without advice regarding timing of protein intake to close proximity of any usual physical activity, on change in physical functioning among community-dwelling older adults with a habitual protein intake of < 1.0 g/kg adjusted (a) BW/d. The design, methods, and primary outcomes of the trial are described in detail elsewhere [27, 28] and are summarized below. A sample of 276 community-dwelling older adults (≥ 65 years) was recruited from the Netherlands and Finland and randomized to one of three groups: (1) intervention group 1 received personalized dietary advice aiming at increasing protein intake to at least 1.2 g/kg aBW/d (*n* = 96); (2) intervention group 2 received personalized dietary advice aiming at increasing protein intake to at least 1.2 g/kg aBW/d *and* advice to optimize the timing of protein intake in close proximity of usual physical activity (*n* = 89); and (3) control group did not receive any intervention (*n* = 91). In addition to being ≥ 65 years and having a habitual protein intake < 1.0 g/kg aBW/d, eligibility criteria included having normal cognition or mild dementia as determined by a Mini-Mental State Examination (MMSE) score > 20 [29], ability to walk 400 m within 15 min, and body mass index (BMI) ≥ 18.5 kg/m^2^ and ≤ 32.0 kg/m^2^. The eligibility criteria described above were assessed during a clinic visit, where body weight and height were measured. Participants also completed a baseline questionnaire, which included questions on demographics (e.g. education), general characteristics (e.g. perceived health), appetite, and other risk factors (e.g. frailty). Randomization was stratified according to participants’ baseline habitual intake (< 0.9 or 0.9–1.0 g/kg aBW/d) and sex across the two countries. The cutoff of 0.9 g/kg aBW/d was assumed to be the mean protein intake among those with low protein intake [30]. A flowchart of the randomization process can be found in Reinders et al. 2021 [28]. For the purposes of this study, only participants from the Netherlands (*n* = 132) are included because the environmental data used is specific to food products consumed in the Netherlands. Further, to assess the effect of the dietary advice aiming at increasing protein intake, the two intervention groups were condensed into one to make comparisons between participants who received dietary advice to increase protein intake (Protein + group, *n* = 84) and those who did not (Control group, *n* = 40). The advice on the timing of protein intake had no effect on food consumption; this was checked by adding both intervention groups as dummy variables to the statistical models and it did not affect the results.

### Intervention: personalized dietary advice to increase protein intake

Trained nutritionists provided participants in the intervention groups with personalized dietary advice to increase protein intake to ≥ 1.2 g/kg aBW/d with isocaloric replacement based on personal habitual dietary characteristics, protein intake and BW of participants as assessed at baseline. Advice was personalized based on food preferences and practices, taking into account whether the participants usually prepared their own meal, where and with whom they ate (e.g. family, friends’ or community home), and whether they typically ate ready-to-eat meals or used a meal service. Participants received both written dietary advice and a verbal explanation from the nutritionist. Advice included the use of habitually consumed protein-rich food products and protein-enriched food products that were not commonly eaten prior to the trial. The protein-enriched food products, which included protein bars, cereals, puddings, coconut whey water and whey powder, were provided for free by the research team and sent to participants’ home as often as needed [28]. The specific products that were sent were uniquely composed based on the participants’ preferences and habitual diet. Guidelines on how to incorporate the protein-enriched food products within their diet were provided. It was also advised to consume at least one daily meal consisting of ≥ 35 g protein to stimulate muscle protein synthesis [31, 32]. Nutritionists provided personalized examples of meals that contain ≥ 35 g protein.

The nutritionists consulted with the participants from the Protein + group several times throughout the intervention period to ensure that they had understood and were able to adhere to the advice. Changes in the dietary advice were made if necessary. For example, participants were requested to contact the nutritionists when a BW change of > 2 kg occurred, so the dietary advice could be adapted accordingly. Participants allocated to the Control group were also contacted at similar time points as the intervention groups to ask how they are doing. To stimulate commitment to the trial, two lectures on non-health-related themes were organized and participants received incentives after three months.

### Dietary data

Food consumption was assessed by three 24-h dietary recalls that were aided by food diaries one week prior to baseline and one week prior to the 3-month and 6-month follow-up clinic visit. The participants were asked to keep track of their dietary intake by filling out a food diary for three consecutive days (three weekdays or two weekdays and one weekend day). They received a diary and booklet with pictures of portion sizes to help them accurately fill out the diary. Nutritionists called the participants to go through their food diary of the day before (24-h dietary recall). In case one of the three days was reported by the participant as not representative, mean intake was based on two instead of three days (*n* = 5).

Food intake data were entered into the program ‘Eetmeter’ of the Netherlands Nutrition Center using an extended version of the Dutch Food Composition Table [33, 34]. Food consumption data were categorized into 20 main food groups, modified from the GloboDiet food group classification, which were used to determine protein-rich food groups for the analysis [35]. Protein-enriched food products provided during the trial were a separate food group, making a total of 21 main food groups (Supplementary Table 1). The energy value provided by protein for each food item was calculated in energy percent (E% protein) [(g protein $x$ (4 kcal / 1 g protein)) / total kcal of the food)] and were averaged across the food items in each food group. Food groups with at least an average 12 E% protein were considered protein-rich, as the European Commission recognizes food with at least 12 E% protein as a source of protein [36]. The ‘Meat and meat products’ food group was further stratified into subcategories due to their different impacts on health and the environment. Further, food groups were classified by protein source category: animal-based, plant-based and miscellaneous. Miscellaneous sources contain both animal and plant-based sources of protein (e.g. meat and dairy substitutes, soups and mixed dishes).

### Environmental data

Various environmental impact indicators were investigated to assess diet sustainability, namely GHGE expressed in kilograms of carbon dioxide equivalents (kg CO_2_-eq), LU in square meter-year (m^2^*y), terrestrial acidification in kg sulfur dioxide equivalents (kg SO_2_-eq), freshwater eutrophication in kg phosphorous equivalents (kg P-eq), marine eutrophication in kg nitrogen equivalents (kg N-eq), and blue water use, representing irrigated water from ground and surface water, in cubic meter (m^3^). The life cycle assessment (LCA) approach was applied to calculate the environmental impact of foods and beverages throughout the entire life cycle, including farming, processing, distribution, through to waste. Primary LCA data was available for 242 foods representative of the Dutch situation and were calculated by Blonk Consultants (Gouda, the Netherlands) for the Netherlands’ National Institute for Public Health and the Environment [37]. An extended dataset including extrapolated data for foods and beverages for which primary data were not available was used [38]. The extended dataset covered 84% of all foods consumed by the trial participants. Additional proxies and extrapolations from the primary data were made for foods and beverages for which data were not available, including protein-enriched food products provided during the trial. Proxies were determined based on similarities in types of food, production systems and ingredient composition by expert judgement. For composite dishes, standardized recipes were used where available, and if not available, recipes were based on label information.

### Statistical analysis

Descriptive statistics were produced to describe the baseline characteristics of the study participants stratified by study group. Education was categorized into three groups, namely lower education (includes elementary education or less), middle education (includes lower vocational education and general intermediate) and higher education (includes intermediate vocational education, general secondary, higher vocational, college or university). Mean daily consumption of protein-rich foods groups and protein source categories (i.e. animal, plant, miscellaneous), GHGEs, LU, blue water use, terrestrial acidification, freshwater eutrophication, and marine eutrophication were calculated for each participant over three days and at each time point (baseline, 3-month, 6-month follow up).

Longitudinal analysis of covariance was carried out using mixed-effects models to assess the effect of dietary advice on food consumption and the environmental impact of the diet. To assess the trial effect on food consumption, protein intake (in grams of protein per day) by protein source (i.e. animal-based, plant-based, and miscellaneous) and consumption of the protein-rich food groups (in grams of food per day) were analyzed. To assess the trial effect on the environmental impact of the diet, GHGE, LU, terrestrial acidification, freshwater and marine eutrophication, and blue water use were analyzed. A random intercept was added to the models to take into account the dependency of the repeated observations among the participants. Participants with missing dietary data at only one of the follow-up measurements were included in the analyses and no imputations were conducted, as a mixed model analysis estimated with the maximum likelihood estimator accounts for missing data [39, 40]. Drop-outs, i.e. participants with missing dietary data at the 3-month follow-up and the 6-month follow-up, were excluded in the analyses (*n* = 8), and therefore the analytical sample included 124 participants.

Adjustment was made for the baseline value of the outcomes to increase precision [39, 40]. Further adjustment was made for sex and baseline energy intake due to group differences at baseline (see Table 1). To explore the effect of the compositional changes on the environmental impact of the diet, secondary analyses were conducted to adjust for energy intake at each time point. Regression coefficients and 95% CI along with p values are presented. For all statistical tests, the 2-sided significance threshold was set to a p-value of 0.05. The analyses were performed on an intention-to-treat basis. Statistical analyses were conducted with Stata 16 (StataCorp. 2019. *Stata Statistical Software: Release 16*. College Station, TX: StataCorp LLC).

**Table 1:** Baseline characteristics of participants who did not receive personalized dietary advice to increase protein intake (Control group) and participants who received personalized dietary advice to increase protein intake (Protein + group) (*N* = 124)

Characteristic	Control group (*n* = 40)	Protein + group (*n* = 84)
Age (y)	74 ± 5	74 ± 4
*Sex*, *n* (%)		
Male	20 (50%)	45 (54%)
Female	20 (50%)	39 (46%)
*Education*^a^, n(%)		
Lower	–	2 (2%)
Middle	13 (32%)	8 (10%)
Higher	27 (68%)	74 (88%)
BMI^b^ (kg/m^2^)	26.7 ± 3	26.2 ± 3
Energy intake (kcal/d)	1678.9 ± 289.3	1759.5 ± 409.6
Protein intake (g/d)	63.2 ± 10.1	62.8 ± 12.2
Protein intake (g/kg aBW/d)	0.83 ± 0.12	0.84 ± 0.14
Greenhouse gas emissions (kg CO_2_-eq/d)	4.1 ± 1.0	4.2 ± 1.3
Land use (m^2^*y/d)	2.5 ± 0.5	2.5 ± 0.7
Terrestrial acidification (kg SO_2_-eq/d)	0.04 ± 0.01	0.04 ± 0.01
Freshwater eutrophication (kg P-eq/d)	3.1 × 10^–4^ ± 7.6 × 10^–5^	3.3 × 10^–4^ ± 1.3 × 10^–4^
Marine eutrophication (kg N-eq/d)	7.4 × 10^–3^ ± 5.0 × 10^–3^	6.5 × 10^–3^ ± 3.1 × 10^–3^
Blue water use (m^3^/d)	0.2 ± 0.1	0.2 ± 0.1

Results are presented in mean ± standard deviation unless reported otherwise

^a^Lower education includes elementary education or less; Middle education includes lower vocational education and general intermediate; Higher education includes intermediate vocational education, general secondary, higher vocational, college or university

^b^Body mass index

## Results

### Study participants and baseline characteristics

Baseline characteristics of the two study groups are presented in Table 1. The participants had an average age of 74 years and an average body mass index of 26 kg/m^2^. The Protein + group had a slightly higher proportion of males and a higher proportion of participants who completed higher education compared to the Control group at baseline. Further, the Protein + group had a higher baseline mean energy intake compared to the Control group. The baseline mean ± sd protein intake of the study participants was 62.9 ± 11.5 g/d or 0.84 ± 0.13 g/kg aBW/d.

### Intervention effect on consumption of protein and protein-rich food groups

The dietary advice aiming at increasing protein intake among community-dwelling older adults led to an increase in protein intake by 46% (95% CI 38–55%) [29.2 g /d (95% CI 23.9–34.5 g/d, *p* < 0.001) or 0.4 g /kg aBW/d (95% CI 0.3–0.5 g/kg aBW/d, *p* < 0.001)] relative to the Control group, when adjusted for sex, baseline energy intake and baseline protein intake. The intervention resulted in more participants reaching or exceeding the recommended protein intake of 1.2 g/kg aBW/d at 6 months: 58% of older adults in the Protein + group compared to 10% in the Control group. Although advice aimed for an isocaloric increase in protein intake, the Protein + group increased their energy intake by 115.3 kcal/d (95% CI 6.9–223.6 kcal/d, *p* = 0.037) from baseline to the 6-month follow-up compared to the Control group. Nevertheless, no difference in mean body weight change was found between the Protein + and Control group (*p* = 0.371). When energy intake at the different time points was taken into account, protein intake increased by 24.7 g/d (95% 19.8–29.7 g/d, *p* < 0.001) in the Protein + group relative to the Control group (results not shown). Furthermore, statistically significant increases in intake of potassium, zinc, and phosphorus were found in the Protein + group compared to the Control group (Supplementary Table 2).

**Fig. 1:**
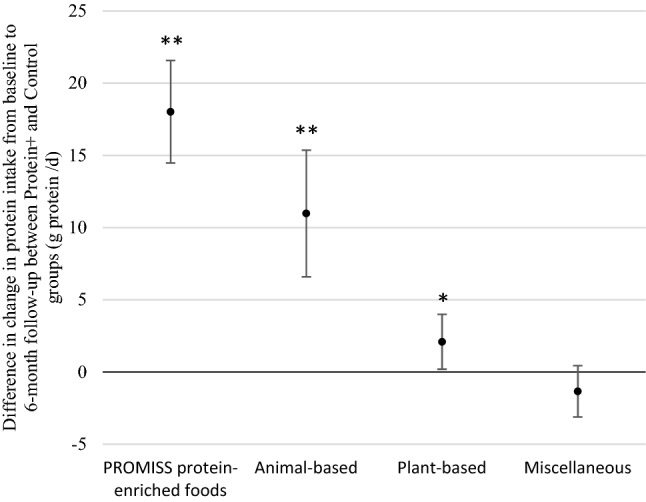
Six-month change in protein intake (in g/d) by protein source among those who received dietary advice aiming at increasing protein intake (Protein + group) compared to those who did not receive dietary advice (Control group) during the PROMISS trial (N = 124), adjusted for sex, baseline energy intake and baseline value of outcome. The dots represent the effect size estimates and lines represent 95% confidence intervals. * p < 0.05 ** p < 0.001

A statistically significant change in protein intake from plant-based and animal-based sources resulted from the dietary advice relative to the control, but no change in protein intake from miscellaneous sources (Fig. 1). The Protein + group increased their consumption of plant-based protein by 2.1 g /d (95% CI 0.2–4.0 g/d, *p* = 0.031) and animal-based protein by 11.0 g /d (95% CI 6.6–15.4 g/d, *p* < 0.001) from baseline to the 6-month follow-up compared to the Control group. Further, the Protein + group increased their consumption of protein from the PROMISS protein-enriched food products by 18 g of protein (95% CI 14.5–21.6 g/d, *p* < 0.001), which were derived from a daily average consumption of 160 g of these products (Table 2). Among the 84 participants in the Protein + group, 84% consumed at least one of the PROMISS protein-enriched food products during the trial. Those who ate the products consumed a daily average 36.3 g of protein bar (8.4 g protein/d, *n* = 26), 39.1 g of cereal crunch (6.5 g protein/d, *n* = 27), 10.8 g protein powder (9.4 g protein/d, *n* = 51), 269.0 g coconut whey water (16.4 g protein/d, *n* = 43), 138.9 g chocolate pudding (14.6 g protein/d, *n* = 9) and 154.2 g vanilla pudding (16.2 g protein/d, *n* = 6). When looking at the relative changes in consumption of the other protein-rich food groups during the trial, only an increase in consumption of milk and milk products was found in the Protein + group compared to the Control group (Table 2).

**Table 2:** Consumption of protein-rich food groups (in g food/d) at baseline and 6-month follow-up of those who did not receive dietary advice (Control group) and those who received dietary advice aiming at increasing protein intake (Protein + group) during the PROMISS trial

	Control group		Protein + group		Difference in change between Protein + and Control group (95% CI)^a^
	Baseline (*n* = 40)	6-month follow-up (*n* = 39)	Baseline (*n* = 84)	6-month follow-up (*n* = 82)	
PROMISS protein-enriched foods^b^	–	–	–	160.0 ± 159.3	157.0* (116.6 – 198.4)
*Animal-based*					
Fish	19.2 ± 26.8	17.6 ± 31.5	22.4 ± 30.5	23.2 ± 34.0	5.2 ( – 6.5 to 17.0)
Meat and meat products	51.2 ± 40.3	71.4 ± 50.5	47.0 ± 41.7	72.3 ± 60.8	8.5 ( – 7.0 to 24.0)
Beef, veal, lamb and goat	20.7 ± 25.9	21.5 ± 25.6	16.5 ± 22.8	25.8 ± 31.1	7.2 ( – 1.4 to 15.8)
Pork	20.2 ± 20.8	33.0 ± 40.1	22.3 ± 29.6	23.6 ± 28.8	0.4 ( – 4.9 to 5.7)
Poultry	10.3 ± 16.8	16.8 ± 33.4	8.3 ± 18.5	21.6 ± 36.8	1.9 ( – 6.1 to 9.9)
Processed meat	13.6 ± 21.2	13.3 ± 21.4	17.4 ± 24.1	16.3 ± 22.8	– 2.8 ( – 9.3 to 3.5)
Eggs	21.1 ± 26.7	21.9 ± 24.4	19.5 ± 20.0	21.3 ± 24.3	2.3 ( – 4.4 to 8.9)
Milk and milk products	275.0 ± 171.5	269.7 ± 166.6	205.8 ± 157.9	287.5 ± 165.4	72.7* (31.9 to 113.6)
Cheese	29.0 ± 21.0	28.7 ± 21.7	28.2 ± 20.0	34.7 ± 26.5	6.7 ( – 0.6 to 14.2)
*Plant-based*					
Vegetables	170.2 ± 103.9	196.0 ± 113.3	184.4 ± 103.6	160.5 ± 88.1	– 14.0 ( – 42.0 to 14.0)
Legumes	5.9 ± 15.0	6.2 ± 15.2	9.5 ± 22.5	10.5 ± 20.1	3.1 ( – 2.0 to 8.1)
Cereal and cereal products	116.4 ± 65.3	119.6 ± 59.3	129.6 ± 67.5	130.8 ± 56.4	8.2 ( – 7.0 to 23.4)
Nuts and seeds	16.4 ± 16.7	14.0 ± 17.1	16.6 ± 21.4	16.5 ± 16.6	2.9 ( – 1.4 – 7.1)
*Miscellaneous*					
Meat and dairy substitutes	2.9 ± 7.8	5.5 ± 14.3	14.6 ± 41.9	16.3 ± 48.6	4.3 ( – 8.9 to 17.5)
Soups	54.1 ± 78.5	59.0 ± 80.7	79.1 ± 99.9	59.6 ± 95.2	– 24.1 ( – 52.5 – 4.3)
Mixed dishes	50.4 ± 117.7	37.7 ± 68.5	41.3 ± 68.3	32.5 ± 58.1	– 20.2 ( – 41.3 to 1.0)

Values displayed as mean ± standard deviation

^a^Unstandardized regression coefficients and 95% confidence intervals of difference in change from baseline to 6-month follow up between Protein + group and Control group, controlling for sex, baseline energy intake and baseline value of outcome

^b^PROMISS protein-enriched food products included protein bars, cereals, puddings, coconut whey water and whey powder. *Statistically significant at *p* < 0.05

### Intervention effect on the environmental impact of diet

The dietary advice aiming at increasing protein intake among community-dwelling older adults led to an increase in GHGE by 0.66 kg CO_2_-eq/d (95% CI 0.29–1.02 kg CO_2_-eq/d), LU by 0.46 m^2^*y/d (95% CI 0.28–0.67 m^2^*y/d), terrestrial acidification by 0.01 kg SO_2_-eq/d (95% CI 0.002–0.01 kg SO_2_-eq/d), and marine eutrophication by 1.04 × 10^–3^ kg N-eq/d (95% CI 7.27 × 10^–5^ to 2.01 × 10^–3^ kg N-eq/d) (Table 3). The dietary advice had no effect on freshwater eutrophication or blue water consumption. Adjustment for energy intake over time attenuated the effect on GHGE (0.40 kg CO_2_-eq/d, 95% CI 0.09–0.71 kg CO_2_-eq/d) and LU (0.30 m^2^*y/d, 95% CI 0.13–0.47 m^2^*y/d) and terrestrial acidification (0.005 kg SO_2_-eq/d, 95% CI 8.69 × 10^–6^ to 0.01 kg SO_2_-eq/d) and no longer had a statistically significant effect on marine eutrophication (7.1 × 10^–4^ kg N-eq/d, 95% CI  – 2.2 × 10^–4^ to 1.6 × 10^–3^ kg N-eq/d).

**Table 3:** Effect of dietary advice aiming at increasing protein intake on the environmental impact of the diet in Dutch community-dwelling older adults during the PROMISS trial (*N* = 124)

Environmental outcomes	Difference in change from baseline to 6 months^a^	95% CI	*P* value
Greenhouse gas emissions (kg CO_2_-eq/d)			
Model 1^b^	0.66	0.29–1.02	< 0.001
Model 2^c^	0.40	0.09–0.71	0.010
Land use (m^2^*y/d)			
Model 1	0.46	0.24–0.67	< 0.001
Model 2	0.30	0.14–0.47	< 0.001
Terrestrial acidification (kg SO_2_-eq/d)			
Model 1	0.01	0.002–0.01	0.010
Model 2	0.01	8.69 × 10^–6^ – 0.01	0.050
Eutrophication—Freshwater (kg P-eq/d)			
Model 1	3.57 × 10^–5^	– 3.37 × 10^–6^ – 7.47 × 10^–6^	0.073
Model 2	1.62 × 10^–5^	– 1.77 × 10^–5^ – 5.01 × 10^–5^	0.349
Eutrophication—Marine (kg N-eq/d)			
Model 1	1.04 × 10^–3^	7.27 × 10^–5^ – 2.01 × 10^–3^	0.035
Model 2	7.11 × 10^–4^	2.19 × 10^–4^ – 1.64 × 10^–3^	0.134
Blue water use (m^3^/d)			
Model 1	– 0.01	– 0.03 to 0.01	0.329
Model 2	– 0.02	– 0.03 to 0.01	0.065

^a^Unstandardized beta coefficient of the difference in change from baseline to 6-month follow-up between participants who received dietary advice (Protein + group) and participants who did not receive dietary advice (Control group)

^b^Model 1 controls for sex, baseline energy intake and baseline value of outcome

^c^Model 2 controls for sex, energy intake over time, and baseline value of outcome

### Contribution of food groups to total protein intake and environmental impacts

At baseline, the top three food groups contributing most to total protein intake across the total study population were cereal products (18% of total protein intake), meat products (16%), and milk products (15%). At the 6-month follow-up, the dominant protein sources in the Protein + group were the PROMISS protein-enriched food products (19% of total protein intake) followed by meat products (17%) and milk products (14%), whereas meat products (23%), cereal products (16%), and milk products (13%) were dominant sources in the Control group (Supplementary Fig. 1 and 2).

For GHGE, LU, terrestrial acidification and marine eutrophication, meat and meat products were the largest contributors at baseline and 6-month follow-up for both groups (Supplementary Fig. 1). Milk and milk products were the second largest contributor to total GHGE, terrestrial acidification, and marine eutrophication at baseline and 6-month follow-up for both groups. The second-largest contributor to LU was drinks (e.g., water, coffee, tea, soft drinks, alcoholic beverages) for both groups except for the Protein + group at the 6-month follow-up, for which the PROMISS protein-enriched food products contributed the most. Drinks contributed most to freshwater eutrophication at baseline, followed by meat and meat products, but the order switched at the 6-month follow-up for both groups. For blue water use, fruits, drinks, and nuts and seeds were the main contributors at baseline and 6-month follow-up for both groups. Among the protein-rich food products, meat and milk products hold the most weight in the diet’s environmental impact across all indicators, except blue water use, for which nuts and seeds hold the most weight.

## Discussion

Dietary advice aiming at increasing protein intake among community-dwelling older adults with lower habitual protein intake (< 1.0 g/kg aBW/d) led to a change in food consumption and an increase in four out of six environmental impact indicators. Older adults who received dietary advice increased their protein intake by 46% compared to older adults who did not receive advice. This result was explained by a small but significant increase in protein intake from plant-based foods, a large significant increase in protein intake from animal-based foods, and an introduction of protein-enriched food products into the diet. These changes made to the diet yielded a significant increase in GHGE by 16%, LU by 19%, terrestrial acidification by 20%, and marine eutrophication by 16% compared to the control group. Once energy intake over time was accounted for, the environmental impacts were attenuated and the trial no longer had an effect on marine eutrophication.

Our findings are consistent with previous studies that modelled theoretical dietary changes from current diets to high-protein diets, which expose a tendency to value animal-based protein, leading to higher environmental impacts. In Switzerland, a hypothetical protein-oriented diet consisted of greater amounts of animal-based foods compared to the current Swiss diet, resulting in a 50% increase in GHGE and a 20% increase in land, nitrogen and phosphorus footprint [18]. Similarly, the diet optimization study among Dutch older adults found that an increase in protein intake from the average intake of 1.0 to 1.2 g/kg BW/d, with no consideration of diet sustainability, led to increases in animal-based protein and an increase GHGE, LU and fossil energy use [20]. The present study shows that in real life, dietary advice aiming at increasing protein intake resulted in increased protein intake mainly from animal-based protein sources, and especially from milk and milk products, and protein-enriched food products. This in turn led to increases in GHGE, LU, terrestrial acidification and marine eutrophication, but no change in freshwater eutrophication and blue water use.

What is unique about this study compared to the aforementioned modelling studies is the inclusion of protein-enriched food products. Protein-enriched food products contributed to approximately one fifth of total protein intake, but to only 5% GHGE, 13% LU, 3% terrestrial acidification, 4% freshwater eutrophication, 3% marine eutrophication, and 2% blue water use in the diet of the Protein + group at the 6-month follow-up. Protein-enriched foods, which are protein-dense given its volume, are thus efficient in delivering protein with relatively low environmental impact. Although consumer studies have shown that older adults tend to be skeptical towards protein-enriched foods [41, 42], this study supports findings from previous trials that show protein-enriched foods are acceptable and can be successfully implemented in the menu of older adults [43–45]. The protein-enriched food product consumed by the most participants was the whey protein powder, which has a relatively high environmental impact being derived from milk. An LCA study shows that 14 kg CO_2_-eq can be avoided by replacing 1 kg whey with 1 kg soy protein [46]. However, whey is a waste product created from cheese making, and therefore its production is inevitable given the high demand for cheese [47]. This creates an opportunity for the dairy industry to channel an environmentally burdensome waste product into protein-enriched foods and beverages for older consumers, although technological innovation is needed to bring down the environmental impact of whey processing and transportation [48].

Evidence is clear that a protein transition is needed to achieve more environmentally-friendly diets [19, 22, 49]. To increase protein intake in an environmentally friendly way in older adults, the diet optimization study showed that a shift towards a more plant-based diet was needed, one in which the animal- to plant-protein ratio shifts from 60:40 to 50:50 [20]. This is in line with the Health Council of the Netherlands’ advice to shift towards a more plant-based diet (i.e. a diet in which 50% of total protein consumed is derived from animal sources and 50% from plant sources) to reconcile the environmental pressures of the current diet, as well as to reduce the risk of chronic diseases associated with high consumption of red and processed meat [50, 51]. When it comes to the transition to plant-based diets, protein quality remains a concern. In general, animal-based proteins are superior to plant-based protein in terms of their higher digestibility and better composition of essential amino acids, but it has been shown that consuming sufficient amounts and a diverse assortment of plant-based foods can provide adequate protein [49]. When it comes to preserving muscle mass among older adults, a higher amount of protein consumed, regardless of protein type, was found to be beneficial, and there was no added value in having a higher animal- to plant-protein ratio [52]. Because a serving of plant-based food contains on average less protein compared to an equivalent portion of animal-based food, more plant-based foods would need to be consumed to obtain sufficient protein intake [53]. This might be problematic for older adults who have physical problems with eating or low appetite who are at higher risk of low protein intake [53, 54]. In this case, protein-enrichment of habitually consumed foods and beverages, in addition to consuming more plant protein in place of animal protein, might support the shift towards environmentally-friendly high-protein diets. Nevertheless, the exclusion of food groups like meat is not necessary to improve the sustainability of diets [20].

This study provides insight into the effect of increasing the current RDA for protein on changes in food consumption and diet sustainability among older adults. Although energy intake and body weight were carefully monitored during the trial, which would not happen if the RDA were to change, the Protein + group increased their energy intake by 115 kcal compared to those in the Control group. However, there was no difference in body weight change between the Protein + and control group [55]. Higher total energy intake, regardless of source, has also been associated with a higher environmental impact on the diet [56, 57], explaining the attenuation of the trial effects on GHGE, LU and terrestrial acidification when energy intake over time was accounted for. Nevertheless, even if energy intake remained constant, the observed increases in GHGE, LU, terrestrial acidification and marine eutrophication serve as a warning. If we are to meet the 2030 GHGE targets of the Intergovernmental Panel on Climate Change (IPCC) report, designed to limit the global average temperature rise to 1.5 °C, the GHGE of the average Dutch diet should be 2.04 kg CO2-eq/d, half of the baseline GHGE estimate [58]. In light of a growing older population and the impending climate crisis, it is necessary to consider environmental sustainability in addition to the nutritional adequacy of the diet in older adults.

The present study has a number of strengths and limitations. A strength of this study was the provision of personalized dietary advice tailored to each participant’s preferences and practices, which is a more effective strategy to change food consumption compared to generalized dietary advice [59]. This allowed us to examine the environmental impact of dietary change within a relatively short period of time (6 months). Further, trained nutritionists performed three 24-h dietary recalls at baseline and two follow-up moments to capture usual protein intake at the population level [60]. The use of the food diaries, which served as a memory aid for participants, might have yielded more accurate recalls since some older adults may have a poorer short-term memory. A drawback to this is that respondents may have unintentionally changed their dietary habits through self-reflection, or intentionally to make their responses socially desirable [61]. A disadvantage to 24-h dietary recalls is their susceptibility to error including misreporting and day-to-day variation, which we did not take into account to establish the distribution of usual dietary intake [62]. It is possible, for instance, that differential response bias due to intervention exposure may have led to over-reporting of protein-rich foods among those in the Protein + group [63]. Day-to-day variation, however, is a random error and is not expected to influence mean intake of the population because on average random errors cancel out, but it may have attenuated the strength of the associations [64]. Another strength is that the LCA data were calculated for each food item from farm to fork using Dutch-specific market mixes (i.e., looking at trade data to take into account the proportion of food imported/produced in country), background processes (e.g., water, electricity, etc.), and food waste data, and therefore are specific to foods consumed in the Netherlands. Such country-specific data that have been collected in a consistent and rigorous manner, however, were not available for Finland. Because country-specific food LCA data are desired given country differences that could arise from varying factors like consumption patterns, climate, topography, and other environmental factors, we chose to exclude participants from Finland. Moreover, the use of five environmental impact indicators gives a more nuanced insight into diet sustainability compared to the majority of studies that focus on GHGE [38]. The environmental impact indicators in our study do not include other metrics like biodiversity loss and antibiotic use in poultry production, due to a lack of robust data. Nevertheless, LCA data have a high level of uncertainty due to various factors such as limited data and variations in local environments [65]. More than 20% of the foods in this study are based on extrapolated data, adding more uncertainty to the environmental estimates [38]. However, the ranking of food groups is unlikely to be affected, and besides, our LCA data is complete [38].

The need for considering environmental sustainability in dietary guidance is clear [66]. Evidence consistently indicates that the impacts of animal-based foods exceed those of plant-based alternatives across multiple environmental indicators including GHGE, LU, acidification, eutrophication, and water use [67, 68]. Nevertheless, there is great variability in impacts between different food products among animal- and plant-based sources. For instance, compared to non-ruminant meat (e.g. poultry, pork), ruminant meat (e.g. beef, goat, lamb) has a much larger environmental impact because ruminants do not efficiently convert feed into bodyweight and they emit methane, a potent greenhouse gas, as a by-product of enteric fermentation during their digestive process [24, 67]. Dietary advice aiming at increasing protein intake among older adults should, therefore, address the proportion of animal- to plant protein in the diet and recommend low-impact alternatives (e.g. poultry vs. beef). More research is warranted to assess the effect of increasing protein intake mainly from plant-based sources such as legumes, nuts, and whole grains with an isocaloric replacement on (long-term) functional outcomes in older adults. In addition, further research is needed to evaluate the environmental impact of dietary change due to dietary advice aiming at increasing protein intake that also considers environmental sustainability.

## Conclusion

Personalized dietary advice aiming at increasing protein intake led to a small increase in protein intake from plant-based sources, a larger increase in protein intake from animal-based sources, and an increase in protein intake from protein-enriched food products. These dietary changes together yielded an increase in GHGE, LU, terrestrial acidification and marine eutrophication, but no change in freshwater eutrophication and blue water use. Once energy intake overtime was accounted for, the intervention no longer had an effect on marine eutrophication. To meet the protein needs of a growing older population, dietary guidance must incorporate environmental sustainability aspects, in particular reducing the animal- to plant-protein ratio and replacing high-impact protein sources with lower-impact protein sources within each protein source category (e.g. poultry to replace beef). Consumers would benefit from receiving clear guidance on how much and what type of foods can sustainably deliver their daily protein needs.

## Supplementary Information

Below is the link to the electronic supplementary material.

Supplementary file1 (DOCX 256 KB)

## Data Availability

Datasets from this research are stored at the repository of the Vrije Universiteit Amsterdam, the Netherlands and potentially available for other researchers after submitting a research proposal.
