# Supplementary material for: Effect of personalized dietary advice to increase protein intake on food consumption and the environmental impact of the diet in community-dwelling older adults: results from the PROMISS trial
Source: Eur J Nutr. 2022 Jul 5;61(8):4015–26. doi: 10.1007/s00394-022-02896-x (PMC9596580; doi:10.1007/s00394-022-02896-x)
Supplement: Supplementary file 1 — Supplementary file1 (DOCX 256 KB) [file 394_2022_2896_MOESM1_ESM.docx]

**Effect of personalized dietary advice to increase protein intake on food consumption and the environmental impact of the diet in community-dwelling older adults: results from the PROMISS trial**

**Authors and affiliations**

Alessandra C. Grasso, Margreet R. Olthof, Ilse Reinders, Hanneke A. H. Wijnhoven, Marjolein Visser, Ingeborg A. Brouwer*

*Department of Health Sciences, Faculty of Science, and the Amsterdam Public Health Research Institute, Vrije Universiteit Amsterdam, Amsterdam, The Netherlands*

^*^ Corresponding author: [ingeborg.brouwer@vu.nl](mailto:ingeborg.brouwer@vu.nl)

**Journal**

European Journal of Nutrition

**Supplementary Table 1** Mean energy content, energy value provided by protein and greenhouse gas emissions (GHGE), land use (LU), terrestrial acidification, freshwater and marine eutrophication, and blue water use per 100g of food groups, based on actual consumption by the Dutch participants of the PROMISS trial^a^

| Protein source category | Main food groups | Sub-food groups | Energy (kcal) | Protein (E%) | GHGE  (kg CO_2_-eq) | LU (m^2^*y/d) | Terrestrial acidification (kg SO_2_-eq/d) | Freshwater eutrophication (kg P-eq/d) | Marine eutrophication (kg N-eq/d) | Blue water use (m^3^/d) |
| --- | --- | --- | --- | --- | --- | --- | --- | --- | --- | --- |
|  | *PROMISS products^b^* |  | *253* | *70* | *0.36* | *0.31* | *0.003* | *2.0x10-5* | *5.9x10-4* | *0.004* |
| Animal-based | | | | | | | | | | |
|  | *Fish and shellfish* |  | *183* | *50* | *0.68* | *0.09* | *0.002* | *8.7x10-5* | *2.6x10-4* | *0.004* |
|  | *Meat and meat products^c^* |  | *227* | *43* | *1.58* | *0.94* | *0.022* | *9.6x10-5* | *3.5x10-3* | *0.015* |
|  |  | *Beef, veal, lamb and goat* | *204* | *55* | *2.93* | *1.46* | *0.052* | *1.3x10-4* | *8.7x10-3* | *0.025* |
|  |  | *Pork* | *201* | *44* | *1.39* | *0.99* | *0.017* | *1.0x10-4* | *2.4x10-3* | *0.013* |
|  |  | *Poultry* | *144* | *68* | *1.00* | *0.68* | *0.008* | *8.2x10-5* | *7.5x10-4* | *0.013* |
|  |  | *Processed meat* | *254* | *35* | *1.39* | *0.89* | *0.019* | *9.2x10-5* | *2.9x10-4* | *0.013* |
|  | *Eggs* |  | *148* | *36* | *0.43* | *0.39* | *0.006* | *4.0x10-5* | *4.8x10-4* | *0.011* |
|  | *Milk and milk products* |  | *89* | *30* | *0.27* | *0.10* | *0.003* | *7.9x10-6* | *4.5x10-4* | *0.002* |
|  | *Cheese* |  | *344* | *28* | *1.15* | *0.50* | *0.016* | *3.8x10-5* | *2.3x10-3* | *0.010* |
| Plant-based | | | | | | | | | | |
|  | *Vegetables^d^* |  | *28* | *24* | *0.16* | *0.05* | *5.9x10-4* | *1.4x10-5* | *1.4x10-4* | *0.007* |
|  | *Legumes* |  | *185* | *22* | *0.37* | *0.05* | *8.6x10-4* | *3.1x10-5* | *3.1x10-4* | *0.007* |
|  | *Cereal and cereal products* |  | *296* | *14* | *0.14* | *0.17* | *0.001* | *1.7x10-5* | *2.9x10-4* | *0.004* |
|  | *Nuts and seeds^e^* |  | *622* | *14* | *0.48* | *0.82* | *0.003* | *7.6x10-5* | *9.2x10-4* | *0.145* |
|  | Potatoes and other tubers |  | 113 | 8 | 0.17 | 0.07 | 0.001 | 1.8x10-5 | 1.8x10-4 | 0.002 |
|  | Fruits |  | 89 | 5 | 0.26 | 0.09 | 0.001 | 1.9x10-5 | 1.5x10-4 | 0.036 |
| Miscellaneous | | | | | | | | | | |
|  | *Meat and dairy substitutes* |  | *208* | *26* | *0.29* | *0.25* | *9.9x10-4* | *1.9x10-5* | *2.2x10-4* | *0.004* |
|  | *Soups* |  | *31* | *18* | *0.14* | *0.06* | *0.001* | *1.0x10-5* | *2.1x10-3* | *0.003* |
|  | *Mixed dishes* |  | *164* | *16* | *0.43* | *0.31* | *0.005* | *3.2x10-5* | *1.0x10-3* | *0.016* |
|  | Condiments and sauces |  | 287 | 8 | 0.36 | 0.33 | 0.002 | 3.5x10-5 | 4.7x10-4 | 0.011 |
|  | Miscellaneous^f^ |  | 198 | 7 | 0.23 | 0.14 | 0.002 | 2.3x10-5 | 3.4x10-4 | 0.004 |
|  | Cakes |  | 413 | 5 | 0.32 | 0.24 | 0.002 | 3.2x10-5 | 5.2x10-4 | 0.012 |
|  | Sugar and confectionary |  | 392 | 2 | 0.26 | 0.28 | 0.002 | 6.7x10-5 | 4.7x10-4 | 0.007 |
|  | Drinks |  | 18 | 1 | 0.05 | 0.03 | 0.003 | 5.8x10-6 | 5.8x10-5 | 0.004 |
|  | Fats |  | 641 | 0 | 0.68 | 0.92 | 0.007 | 5.6x10-5 | 1.3x10-3 | 0.056 |

^a^ Protein-rich food groups, italicized, have at least 12 energy percent (E%) of protein. ^b^ PROMISS protein-enriched food products included protein bars, cereals, puddings, cocowhey water and whey powder. ^c^ Meat and meat products includes the subcategories listed in the table (3.1-3.4) plus offal. ^d^ Vegetables include pod vegetables such as peas, broad beans, string beans and green beans. ^e^ Nuts and seeds include peanuts and peanut butter. ^e^ Miscellaneous food items include unclassified food products and snacks.

**Supplementary Table 2** Daily nutrient intake at baseline and 6-month follow-up of those who did not receive dietary advice (Control group) and those who received dietary advice aiming at increasing protein intake (Protein+ group) during the PROMISS trial

|  | Control group | | Protein+ group | | Difference in change between Protein+ and Control group (95% CI)^a^ |
| --- | --- | --- | --- | --- | --- |
|  | Baseline  (n=40) | 6-month follow-up (n=39) | Baseline  (n=84) | 6-month follow-up (n=82) |  |
| Energy (kcal) | 1678.9 + 289.3 | 1717.3 + 343.7 | 1759.496 + 409.61 | 1872.7 + 436.0 | 115.3 (6.9 – 223.6) * |
| Fat (g) | 71.5 + 18.2 | 73.0 + 18.0 | 71.84 + 21.43 | 73.1 + 24.0 | 0.1 (-6.3 – 6.5) |
| Saturated fat (g) | 27.4 + 7.9 | 28.2 + 9.4 | 26.5 + 10.0 | 28.0 + 11.4 | -0.3 (-3.3 – 2.7) |
| Carbohydrates (g) | 173.7 + 47.7 | 174.4 + 52.4 | 181.3 + 181.3 | 182.5 + 54.3 | 2.4 (-9.9 – 14.8) |
| Total sugars (g) | 84.1 + 30.8 | 83.0 + 34.8 | 81.0 + 30.2 | 81.4 + 31.1 | 1.7 (-5.4 – 8.8) |
| Protein (g) | 63.2 + 10.1 | 67.0 + 14.0 | 62.8 + 12.2 | 94.8 + 21.4 | 29.2 (23.9 – 34.5) ** |
| Protein (g/adjusted kg body weight) | 0.83 + 0.11 | 0.88 + 0.19 | 0.84 + 0.14 | 1.27 + 0.26 | 0.4 (0.3 – 0.5) ** |
| Fiber (g) | 21.5 + 14.2 | 19.8 + 5.6 | 21.7 + 6.7 | 21.3 + 6.2 | 1.7 (-0.04 – 3.5) |
| Sodium (g) | 1859.2 + 663.9 | 1906.2 + 641.3 | 2004.7 + 759.2 | 2077.7 + 770.8 | 83.6 (-117.4 – 284.5) |
| Potassium | 2879.0 + 572.4 | 2966.6 + 625.6 | 3015.7 + 738.7 | 3138.0 + 984.5 | 72.5 (-147.8 – 292.9) |
| Calcium | 910.0 + 216.01 | 873.6 + 283.4 | 844.7 + 289.7 | 1028.3 + 306.8 | 189.5 (92.3 – 286.6) ** |
| Magnesium | 299 + 69.6 | 292.9 + 68.7 | 311.5 + 91.2 | 327.7 + 94.4 | 23.4 (-0.7 – 47.7) |
| Iron | 9.0 + 2.1 | 9.3 + 2.3 | 9.6 + 2.6 | 11.5 + 4.7 | 1.3 (0.4 – 2.2) * |
| Selenium | 45.6 + 24.8 | 47.8 + 39.9 | 44.3 + 22.4 | 48.0 + 21.1 | -4.3 (-14.6 – 6.1) |
| Zinc | 8.4 + 1.6 | 8.8 + 2.2 | 8.3 + 1.9 | 9.7 + 2.6 | 1.1 (0.4 – 1.8) * |
| Vitamin A | 694.5 + 389.0 | 815.6 + 568.5 | 836.9 + 546.3 | 954.3 + 1250.9 | -2.0 (-292.2 – 288.1) |
| Vitamin D | 2.7 + 1.7 | 2.7 + 2.5 | 2.8 + 1.8 | 3.2 + 2.2 | 1.2 (-0.8 – 3.2) |
| Vitamin E | 9.7 + 2.7 | 9.7 + 2.8 | 10.6 + 4.8 | 10.3 + 3.4 | 0.3 (-0.7 – 1.3) |
| Vitamin B1 | 0.8 + 0.2 | 0.9 + 0.3 | 0.8 + 0.3 | 0.9 + 0.3 | 0.02 (-0.06 – 0.11) |
| Vitamin B2 | 8.8 + 47.9 | 1.3 + 0.4 | 2.6 + 12.7 | 1.6 + 1.5 | -0.1 (-2.0 – 1.8) |
| Niacin | 13.1 + 3.1 | 13.9 + 4.1 | 13.8 + 4.3 | 15.9 + 6.8 | 1.1 (-0.5 – 2.7) |
| Vitamin B6 | 1.2 + 0.3 | 1.3 + 0.4 | 1.3 + 0.5 | 1.4 + 1.4 | -0.03 (-0.3 – 0.3) |
| Folate | 237.0 + 78.8 | 259.0 + 96.5 | 266.3 + 110.3 | 269.9 + 90.7 | -1.5 (-37.8 – 34.7) |
| Vitamin B12 | 3.7 + 1.9 | 3.8 + 1.8 | 4.1 + 2.9 | 5.7 + 7.6 | 1.8 (0.1 – 3.4) |
| Vitamin C | 93.5 + 48.0 | 99.5 + 48.5 | 103.6 + 49.1 | 97.8 + 53.6 | -4.6 (-19.2 – 9.9) |
| Iodine | 135.2 + 39.5 | 138.5 + 47.5 | 147.2 + 52.2 | 154.6 + 67.2 | 15.2 (-15.9 – 46.3) |
| Phosphorus | 1210.3 + 212.4 | 1227.3 + 272.6 | 1181.5 + 281.6 | 1380.9 + 329.6 | 183.3 (99.7 – 267.0) ** |

Values displayed as mean + standard deviation. ^a^ Unstandardized regression coefficients and 95% confidence intervals of difference in change from baseline to 6-month follow up between Protein+ group and Control group, controlling for sex, baseline energy intake and baseline value of outcome. * Statistically significant at p<0.05 **p<0.001.

| a) | b) |
| --- | --- |
| c) | d) |
| e) | f) |
| g) | |

**Supplementary Figure 1** Average (a) protein intake in grams protein per day, (b) greenhouse gas emissions in kg CO_2_-eq per day, (c) land use in m^2^*y per day, (d) terrestrial acidification in kg SO_2_-eq per day, (e) freshwater eutrophication in kg P-eq per day, (f) marine eutrophication in kg N-eq per day, (g) blue water use in m^3^ per day by those who received dietary advice aiming at increasing protein intake (Protein+ group) and those who did not (Control group) at baseline and at the 6-month follow-up of the PROMISS trial

| a) | b) |
| --- | --- |
| c) | d) |
| e) | f) |
| g) | |

**Supplementary Figure 2** Percent contribution of protein source to (a) protein intake in grams protein per day, (b) greenhouse gas emissions in kg CO_2_-eq per day, (c) land use in m^2^*y per day, (d) terrestrial acidification in kg SO_2_-eq per day, (e) freshwater eutrophication in kg P-eq per day, (f) marine eutrophication in kg N-eq per day, (g) blue water use in m^3^ per day by those who received dietary advice aiming at increasing protein intake (Protein+ group) and those who did not (Control group) at baseline and at the 6-month follow-up of the PROMISS trial
